# Coconut (*Cocos nucifera* L.) sap as a potential source of sugar: Antioxidant and nutritional properties

**DOI:** 10.1002/fsn3.1191

**Published:** 2019-09-30

**Authors:** Muhammad Tuseef Asghar, Yus Aniza Yusof, Mohd. Noriznan Mokhtar, Mohammad Effendy Ya'acob, Hasanah Mohd. Ghazali, Lee Sin Chang, Yanty Noorzianna Manaf

**Affiliations:** ^1^ Department of Farm Machinery and Power University of Agriculture Faisalabad Pakistan; ^2^ Department of Process and Food Engineering Faculty of Engineering Universiti Putra Malaysia Serdang Malaysia; ^3^ Laboratory of Halal Services Halal Products Research Institute Universiti Putra Malaysia Serdang Malaysia; ^4^ Department of Food Science Faculty of Food Science and Technology Universiti Putra Malaysia Serdang Malaysia

**Keywords:** antioxidant properties, coconut sap, mineral content, proximate composition, sugar palm juice, sugar profile, sugarcane juice, vitamin profile

## Abstract

This study was carried out to compare the antioxidant and nutritional properties of coconut (*Cocos nucifera* L.) sap with other natural sources of sugar such as sugar palm (*Borassus flabellifer*) and sugarcane (*Saccharum officinarum* L.). Coconut sap and juice from sugar palm and sugarcane were analyzed for proximate composition, pH and total soluble solid (TSS), color, sugar profile, vitamin profile, antioxidant properties (total phenolic contents, DPPH, FRAP, and ABTS), and mineral content. The results indicated that coconut sap possesses high DPPH (23.42%), FRAP (2.09 mM/ml), and ABTS (21.85%) compared with the juices. Coconut sap also had high vitamin C (116.19 µg/ml) and ash (0.27%) contents, especially in potassium (960.87 mg/L) and sodium (183.21 mg/L) which also indicating high content of minerals. These properties showed that coconut sap could be served as a potential healthier sugar source compared with sugar palm and sugarcane juices.

## Graphical abstract

Coconut sap could be served as a potential healthier sugar source compared with sugar palm and sugarcane juices as it carries more minerals, antioxidants, and vitamins.

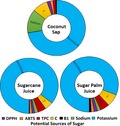

## 1 INTRODUCTION

Increasing threats of diabetes, obesity, hypertension, and heart diseases have become real concerns for many people due to high consumption of sugar in food, beverage, and confectionery products (Chattopadhyay, Raychaudhuri, & Chakraborty, 2014). According to the World Health Organization (WHO, 2018), 451 million of people are living with diabetes and about 43% of total deaths under the age of 70 are diabetic patients. The prevalence of diabetes, overweight, obesity, and physical inactivity in Malaysia is 16.9%, 37.3%, 12.9%, and 51.6%, respectively, and about 3% of total deaths are caused by diabetes (Cho et al., 2018; WHO, 2018). Nowadays, low‐sugar, sugar‐free, and synthetic sugar products are abundantly available in food markets. However, many of these are considered unsafe and unhealthy, as these products can cause side effects such as weight‐gain, brain tumor, and balder cancer (Kroger, Meister, & Kava, 2006; Sharma, Amarnath, Thulasimani, & Ramaswamy, 2016). Thus, this issue imposes an urgent need for the development of healthier sugar products.

Production of natural sweeteners with low glycemic index (GI) can be a solution to diabetes problem. GI is defined as a system (ranks 0–100) which is used to measure blood glucose raised after the consumption of carbohydrate‐containing foods. A food with low GI raises blood glucose less than that of the food with high GI; thus, a healthy product is always associated with low GI value (ADA, 2018). Coconut (*Cocos nucifera* L.) sugar was reported to have a GI value of 35 (Kusumawaty, Maharani, & Edwina, 2012; Trinidad, Mallillin, Sagum, & Encabo, 2010) while the sugar from palm sugar (*Borassus flabellifer*) and sugarcane (*Saccharum officinarum* L.) has GI values of 42 and 58–82, respectively (Saputro et al., 2017). The lower GI value of coconut sugar suggests that it can be a better source of healthier sugar. Coconut sugar is made by evaporation of coconut sap and is a nutrient‐rich crystalline sugar/sweetener that looks, tastes, dissolves, and melts almost exactly the same as regular sugar, but it is completely natural and unrefined and has a far superior taste (Abdullah et al., 2014). The texture and flavor of coconut sugar are also similar to those of brown sugar (Appetit, 2018; Beck, 2014). Thus, it can easily replace regular table sugar.

The coconut tree is widely grown in tropical regions, especially in South Asia, Africa, South America, Australia, and other tropical countries (Morton, 1988), and it is an important source of a refreshing drink called “coconut water” Watawana, Jayawardena, Gunawardhana, and Waisundara (2016). A coconut tree produces inflorescence throughout the year, and coconut sap is collected from unopened spadix of the coconut tree (Ghosh, Bandyopadhyay, Das, Hebbar, & Biswas, 2018; Hebbar et al., 2015). Kusumawaty et al. (2012) and Ysidor et al. (2015) reported that coconut sap records a greater economic value when used as nonfermented and fermented drinks, alcoholic beverages, vinegar, and acetic acid, etc (Ghosh et al., 2018; Ysidor et al., 2015). It is also used as a raw material for the production of coconut sugar. Barh and Mazumdar (2008) found that coconut sap is the richest source of nutrients compared with those of sugar palm juice and date palm sap. However, coconut sap is very susceptible to natural fermentation (Hebbar et al., 2015). Hence, it should be kept at low temperature (−2 ± 1°C) or should be processed immediately to preserve its nutrients.

Sugar palm is a well‐known tropical plant found in India, Thailand, Seri Lanka, Myanmar, Cambodia, Indonesia, and Malaysia (Victor, 2015). Sugar palm juice is oyster white in color, has a neutral pH, and is rich in sugar (10%–15%). It is widely used as fresh drink, fermented to produce alcoholic beverages such as toddy, wine, and arak, ethanol, and as a raw material for sugar (syrup, cake and powder) production (Hebbar et al., 2018; Naknaen & Meenune, 2015; Naknean, 2010). Meanwhile, sugarcane is known as important commercial crop and is recognized as a source of sugar, jaggery (a semi refined sugar mostly used in India and Pakistan), and ethanol (Nath, Dutta, Kumar, & Singh, 2015; Nguyen, Harifara, & Shiro, 2016). It is also used as a fodder for feeding livestock, and its by‐products are used in board making (Miller & Raczuk, 1969).

Based on the above, this study was conducted to compare the antioxidant and nutritional properties of coconut sap as a potential source of healthy sugar with those of sugar palm and sugarcane juices. The investigation is expected to reveal the properties of the sap and juices and their benefits for low GI sugar production, food product development, and the pharmaceutical industry.

## 2 MATERIALS AND METHODS

### 2.1 Materials

Coconut sap and sugar palm juice were purchased fresh from a coconut farm located at Jelai (2.781622, 102.4323312), Taman Bahau, Negeri Sembilan, and Malaysia. Sugarcane juice was purchased fresh from a local wet market in Seri Kembangan, Selangor, and Malaysia. All samples were stored in an ice box and immediately transported back to the Unit Operations Laboratory, Department of Process and Food Engineering, Faculty of Engineering. The sap and juices were filtered through a piece of cheese cloth and stored in a chiller at 4 ± 2°C prior to analysis.

### 2.2 Analysis of sap and juices

#### 2.2.1 Proximate composition

Proximate analysis was performed using a standard procedure of AOAC for moisture, ash, and crude fat contents (AOAC, 2000).

For moisture analysis, about 5 g of sample was weighed into a predried and preweighed crucible and placed in a vacuum oven (VD23, Binder GmbH). The temperature was maintained at 70 ± 5°C and 90 ± 10 mbar pressure for overnight in order to obtain a constant mass. The sample was cooled in a desiccator and the change in weight was determined using an analytical balance (XSE 204, Mettler Toledo). The moisture content was calculated using Equation 1.

(1) $$\frac{\left(\text{Initial}\;\text{weight}\;\text{of}\;\text{dish}\;\text{with}\;\text{sample}-\text{Final}\;\text{weight}\;\text{of}\;\text{dish}\;\text{with}\;\text{sample}\right)}{\left(\text{Initial}\;\text{weight}\;\text{of}\;\text{dish}\;\text{with}\;\text{sample}-\text{Initial}\;\text{weight}\;\text{of}\;\text{empty}\;\text{dish}\right)}\times 100=\%\;\text{MC}$$

For ash analysis, about 2 g of sample was weighed into a predried and preweighed crucible. The crucibles were placed in a muffle furnace (MTI Corporation) and burned at 550°C until white ash was obtained. Then, the sample was cooled in a desiccator and weighed using an analytical balance (XSE 204, Mettler Toledo). The ash content was calculated using Equation 2.

(2) $$\frac{\left(\text{Weight}\;\text{of}\;\text{ash}\right)}{\left(\text{Weight}\;\text{of}\;\text{sample}\right)}\times 100=\%\;\text{Ash}$$

Crude fat content was determined using a Soxhlet extraction apparatus (Soxtlet™ 2050: Foss Analytical). Approximately, 2 g of dried coconut sap, sugar palm juice, and sugarcane juice was weighed into separate thimbles and about 80 ml of extraction solvent (petroleum ether, boiling point, bp = 40–60°C) was filled in predried and preweighed aluminum cups. The thimbles were tightly plugged, and the Soxhlet apparatus was assembled. The apparatus was allowed to reflux for 75 min. After completion of extraction, the extracted material in the cup was dried in an oven (OF‐G22W; Jeio Tech) for 1 hr and then cooled in a desiccator until a constant weight was obtained. The percentage of fat content was obtained using Equation 3.

(3) $$\frac{\left(\text{Weight}\;\text{of}\;\text{fat}\right)}{\left(\text{Weight}\;\text{of}\;\text{sample}\right)}\times 100=\%\;\text{Fat}$$

Crude protein was determined according to the micro‐Kjeldahl method (AOAC, 2012). Crude fiber was not determined in this study as previous literature found that the value was negligible (Barh & Mazumdar, 2008; Naknean & Meenune, 2011; Victor, 2015).

#### 2.2.2 pH and total soluble solids (TSS)

The pH of coconut sap, sugar palm juice, and sugarcane juice was determined using a pH meter (Milwaukee pH‐600) at room temperature (25 ± 1°C). Calibration was accomplished employing pH 4 and 7 buffer solutions.

Total soluble solids (TSS) was measured using an ATC‐Handheld °Brix Refractometer (RHB‐90ATC) with a wide TSS measuring range (0%–90%). One to two drops of samples (original with no dilution) were spread on the glass prism, and °Brix value was read at room temperature (25 ± 1°C; Magwaza & Opara, 2015).

#### 2.2.3 Color analysis

Color measurement of the coconut sap and juice samples was carried out using a handheld portable colorimeter (CR 400, Minolta Co.). Instrumental color data were provided in accordance with the CIE system in terms of L* (lightness and darkness), a* (redness and greenness), and b* (yellowness and blueness). These components were also used for determination of hue angle (H*) which indicates how human eyes see the color and chroma (C*) which indicates the purity of color (Naidu et al., 2016). H* and C* values were calculated using Equations 4 and 5, respectively.

(4) $$C^{*}=\sqrt{a^{*2}+b^{*2}}$$

(5) $$H^{*}=\arctan\left(\frac{b^{*}}{a^{*}}\right)$$

Browning index (BI) was determined using the following Equation 6 (Subhashree, Sunoj, Xue, & Bora, 2017).

(6) $$\text{BI}=\frac{100\left(x-0.31\right)}{0.17}$$

where *x* is

(7) $$x=\frac{a^{*}+175L^{*}}{5.645L^{*}+a^{*}-3.012b^{*}}$$

#### 2.2.4 Sugar profile

Sugar profiling was performed using a high‐performance liquid chromatography (HPLC) comprised of Waters Alliance 2695 separation module (Waters Corporation) equipped with a refractive index detector (RID; Waters 2414 Corporation). Coconut sap, sugar palm, and sugarcane juices were diluted 10 times with deionised water and then filtered using a 0.45 µm nylon filter (Labserve). Twenty microliters of sample were injected into a LiChroCART^®^ Single bond NH_2_ column (Merck) with dimensions of 250 mm × 4.6 mm, particle size of 5 µm. The temperature of the column was set at 40°C. The mobile phase consisted of HPLC‐grade acetonitrile and double‐distilled water (80:20, v/v ratio; Chang, Karim, Mohammed, & Ghazali, 2018). The sugars were separated isocratically at a flow rate of 1.5 ml/min. Standard curves were constructed based on sugar reference standards (fructose, glucose, and sucrose) by plotting peak area against various concentrations of each sugar (0%–5% w/v; Chang et al., 2018; Hunt, Jackson, Mortlock, & Kirk, 1977).

#### 2.2.5 Vitamin profile

Vitamin profiling was performed using a high‐performance liquid chromatography (HPLC) using a Shimadzu liquid chromatograph LC‐10*vp* fitted with a UV‐VIS detector (SPD‐10A, *vp*) set at 210 nm. The samples were first diluted 10 times with deionised water and then filtered using a 0.45 µm nylon filter. Sample (10 µl) was injected into a C18 column (Poroshell 120 EC, Agilent) with dimensions of 100 × 4.6 mm and particle size of 4 µm. The temperature of the column was set at 20°C. Potassium dihydrogen phosphate buffer (50 mM, pH 3.4) was used as the mobile phase and was prepared by mixing 3.40 g of potassium dihydrogen phosphate (KH_2_PO_4_) with 0.1% orthophosphoric acid (H_3_PO_4_) solution in a 500 ml volumetric flask until the marked level. The mobile phase was then filtered through a 0.45 µm nylon filter. The vitamins were separated isocratically at a flow rate of 1.0 ml/min. A set of external vitamin standards (vitamins B1, B2, B3, B4, B10, and C; Merck) was used for the identification of the sample peaks. The quantitation was achieved based on peak area of each vitamin. Calibration curve of analyst was constructed by plotting peak areas versus various concentrations of each vitamin (10–165 ppm).

### 2.3 Antioxidants activity

#### 2.3.1 DPPH method

DPPH scavenging activity of coconut sap, sugar palm juice, and sugarcane juice was determined according to Phisut and Jiraporn (2013) with some modifications. A sample (50 µl) was placed into 96‐wells microplate in triplicate, and 195 µl of 0.2 mM 2,2‐diphenyl‐1‐picrylhydrzyl (DPPH) was added. Double‐distilled water (50 µl) added with 195 µl of DPPH was used as a control. The mixture was gently swirled for 1 min and allowed to stand in the dark at 25 ± 5°C for 60 min. The absorbance of samples was measured at 517 nm using a spectrophotometer (Benchmark Plus Microplate, Bio‐RAD 170‐6930). Different concentrations of Trolox (Merck) in distilled water were used to construct a standard curve. The scavenging activity was calculated from following Equation 8 obtained by regression analysis of standard curve.

(8) $$\%\;\text{Antioxidant}\;\text{activity}=\frac{\text{Abs}\left(\text{control}\right)-\text{Abs}\left(\text{sample}\right)}{\text{Abs}\left(\text{sample}\right)}\times 100$$

where

Abs(control) = Absorbance of solvent (double‐distilled water).

Abs(sample) = Absorbance of sample and standard.

#### 2.3.2 ABTS method

The ABTS (2,2′‐azino‐bis‐3‐ethylbenzthiazoline‐6‐6‐sulfonic acid) method was used as described by Biskup, Golonka, Gamian, and Sroka (2013) with slight modifications using a T‐AOC assay kit (E‐BC‐K219, Elabscience^®^). The required amount of ABTS working solution was prepared by mixing glycine buffer (pH 4.5) (Reagent 1), diammonium 2,2′‐azino‐bis(3‐ethylbenzothiazoline‐6‐sulfonate) (Reagent 2), and hydrogen peroxide (Reagent 3) at a ratio of 76:5:4 and stored in the dark at 25 ± 5°C. Reagent 4 was prepared by mixing peroxide and double‐distilled water in a ratio of 1:9. A standard solution was prepared with different concentrations (0.10, 0.20, 0.40, 0.80, and 1.00 mM) of 10 mM of Trolox (Reagent 5) (Su et al., 2007). The required amount (10 µl) of sample (coconut sap, sugar palm juice, and sugarcane juice), solvent (double‐distilled water; 10 µl), and standard solutions (10 µl) was placed in a microtitre plate in triplicate and mixed with 20 µl of Reagent 4. Then, 170 µl of the ABTS working solution was added and the mixture was allowed to react at room temperature (25 ± 5°C) for 6 min. The absorbance was then measured using a spectrophotometer (Benchmark Plus Microplate, Bio‐RAD 170‐6930) at 405 nm. A standard curve was plotted using absorbance values over different concentrations of standard solution to calculate antioxidant capacity.

#### 2.3.3 FRAP method

Ferric Reducing Antioxidant Power (FRAP) method was used as described by (Benzie & Strain, 1996) with a slight modification using a T‐AOC assay kit (E‐BC‐K225). The required amount of FRAP working solution was prepared by mixing acetic acid buffer (pH 3.6), (2,4,6‐tris (2‐pyridyl) triazine) and iron trichloride at a ratio of 10:1:1. The FRAP solution was then stored in a dark room at 37°C and was used within 2 hr. A standard solution was prepared by mixing 27.8 mg of Fe_2_SO_4_‐7H_2_O with double‐distilled water to a final volume of 1 ml. Then, the standard solutions were diluted in distilled water with different concentrations of 0.15, 0.30, 0.60, 0.90, 1.20, and 1.50 mM. The required amount (5 µl) of sample (coconut sap, sugar palm juice, and sugarcane juice), solvent (double‐distilled water; 5 µl), and standard solutions (5 µl) was pipetted in a 96‐wells microtitre plate in triplicate and mixed with 180 µl of preprepared FRAP solution and allowed to react for 5 min at 37°C. Then, the absorbance was measured at 593 nm using a spectrophotometer (Benchmark Plus Microplate, Bio‐RAD 170‐6930). A standard curve was plotted using absorbance values on *y*‐axis and concentration values on *x*‐axis to calculate the total antioxidant capacity.

### 2.4 Total phenolic contents

The total phenolic compound content was evaluated using Folin‐Ciocalteau Regent (FCR) according to the method described by Karseno, Yanto, Setyowati, and Haryanti (2018). The required amount (10 µl) of sample (coconut sap, sugar palm juice, and sugarcane juice), double‐distilled water (10 µl), and standard solutions (10 µl) was pipetted in a 96‐wells microtiter plate in triplicate. The sample was mixed with 100 µl of 0.2 N FCR and 80 µl of 7.5% Na_2_CO_3_ solution. The mixture was stored for 90 min in an incubator at 30°C. Standard solution was prepared with different concentrations (0, 50, 100, 150, 250, and 500) from gallic acid stock solution (0.5 g gallic acid + 10 ml ethanol, top up with dH_2_O in a 100 ml flask). Spectrophotometer (Benchmark Plus Microplate, Bio‐RAD 170‐6930) was used to measure the absorbance at wavelength of 765 nm. A standard curve was plotted using absorbance values of the standard over different concentrations, and results were expressed as garlic acid equivalent (GAE; Gupta, 2015).

### 2.5 Mineral analysis

Minerals in the form of macro and micronutrients such as calcium (Ca), magnesium (Mg), manganese (Mn), copper (Cu), sodium (Na), potassium (K), zinc (Zn), and iron (Fe) were analyzed according to the method of Shafie, Aris, and Haris (2014) using a N_2_O/acetylene flame atomic absorption spectrophotometer (FAAS; Shimadzu AA‐6800F, Shimadzu Corporation). Coconut sap, sugar palm juice, and sugarcane juice were diluted 10×, 50×, 100×, 500×, and 1,000× in deionised and filtered through a 0.45 µm nylon filter. The samples (15 ml in test tubes) were installed in an automatic injection tray, and sample volume (100 µl) was injected to AAS for analysis of minerals and Lumina cathodes lamp was used for each element. The concentrations of elements were determined from standard curves developed from standard solutions of respective elements.

### 2.6 Statistical analysis

The significance of variations within three samples (coconut sap, sugar palm, and sugarcane juices) was analyzed using one‐way analysis of variance (ANOVA) using SPSS Statistics v 21.0 software (IBM). The values are the mean of three experiments. The means values presented were separated using Duncan's Multiple Range Test (DMRT) at confidence level of 95% (*p* ≤ .05).

## 3 RESULTS AND DISCUSSION

### 3.1 Proximate composition

The proximate composition of coconut sap, sugar palm juice, and sugarcane juice presented in Table 1. The moisture content of coconut sap was 85.93 ± 0.66%, which was slightly higher than the value (85.24%) reported by Ho, Aida, Maskat, and Osman (2008). Meanwhile, the ash, crude fat, and crude protein of coconut sap were 0.27 ± 0.03%, 0.01 ± 0.00%, and 0.26 ± 0.02%, respectively, which was similar to the values reported by Bipasa (2016). As stated previously, crude fiber was not determined in this study as previous findings showed that coconut sap (Barh & Mazumdar, 2008) and sugar palm juice (Naknean & Meenune, 2011; Victor, 2015) contained a very low amount of crude fiber.

**Table 1:** Proximate composition, pH and TSS, and color parameters for coconut sap, sugar palm juice, and sugarcane juice

Attribute	Coconut sap	Sugar palm juice	Sugarcane juice
Moisture content (%)	85.93 ± 0.66^a^	85.77 ± 1.17^a^	84.15 ± 0.90^b^
Ash content (%)	0.27 ± 0.03^a^	0.25 ± 0.02^a^	0.17 ± 0.02^b^
Crude fat (%)	0.01 ± 0.00^b^	0.03 ± 0.00^b^	0.40 ± 0.07^a^
Protein (%)	0.26 ± 0.02^b^	0.27 ± 0.02^b^	0.44 ± 0.03^a^
Carbohydrate (%)	13.53 ± 0.64^b^	14.68 ± 1.13^b^	16.84 ± 0.85^a^
pH	5.52 ± 0.08^a^	4.92 ± 0.07^b^	4.51 ± 0.08^c^
TSS (%)	12.40 ± 1.14^b^	13.00 ± 1.58^b^	14.40 ± 1.82^a^
Color			
L*	38.16 ± 0.63^a^	32.52 ± 0.75^b^	31.10 ± 034^c^
a*	−0.92 ± 0.10^b^	−0.80 ± 0.08^b^	0.68 ± 0.13^a^
b*	−3.5 ± 0.04^b^	−4.29 ± 0.17^c^	4.97 ± 0.18^a^
C*	3.62 ± 0.07^c^	4.37 ± 0.16^b^	5.02 ± 0.16^a^
H*	1.31 ± 0.02^c^	1.38 ± 0.02^b^	1.43 ± 0.03^a^
Browning index	−10.21 ± 0.29^b^	−13.71 ± 0.56^c^	18.65 ± 0.31^a^

Note

Values in the same row sharing different small letters (a, b, c) are expressed as significantly different (*p* ≤ 0.05) among the samples.

Coconut sap had a high moisture (85.93%) and ash (0.27%) contents as compared to sugar palm and sugarcane juice (0.25% and 0.17%, respectively). A high ash content indicates that coconut sap contained more minerals. Therefore, processing of coconut sap into sugar could be used to produce a mineral‐rich sugar (Hebbar et al., 2015). On the other hand, the amount of crude fat in coconut sap (0.01%) and sugar palm juice (0.03%) was significantly (*p* ≤ .05) lower than sugarcane juice (0.40%), which may lead to the production of healthier sugar from coconut. The value of carbohydrate from coconut sap was the lowest (13.53%) as compared to those of sugar palm (14.68%) and sugarcane juices (16.84%), indicating coconut sap was a carbohydrate‐rich source.

### 3.2 pH and total soluble solid (TSS)

pH of coconut sap used in this study was 5.52 ± 0.08 (Table 1), indicating the sap had low acidity. The result obtained was different with a finding reported by Hebbar et al. (2015) (pH = 4.5), possibly due to varietal differences. The pH value of coconut sap was significantly (*p* ≤ .05) higher than those of sugar palm and sugarcane juices, indicating coconut sap was less acidic as compared to sugar palm and sugarcane juices.

The TSS content of coconut sap was found to be 12.40 ± 1.14°Brix (Table 1) which was in the range of 12–14°Brix as reported by Bipasa (2016) and Hebbar et al. (2015). The TSS value of sugarcane juice (14.40°Brix) was also in the range of 14–16°Brix as reported by Xiao, Liao, and Guo (2017). Overall, the TSS of coconut sap was the lowest as compared to sugar palm and sugarcane juices. However, there is no significant difference (*p* > .05) in TSS for coconut sap and sugar palm juice but both are significantly (*p* ≤ .05) lower than sugarcane juice, indicating coconut sap had lower soluble matter and contained less sugar.

### 3.3 Color

**Figure 1:**
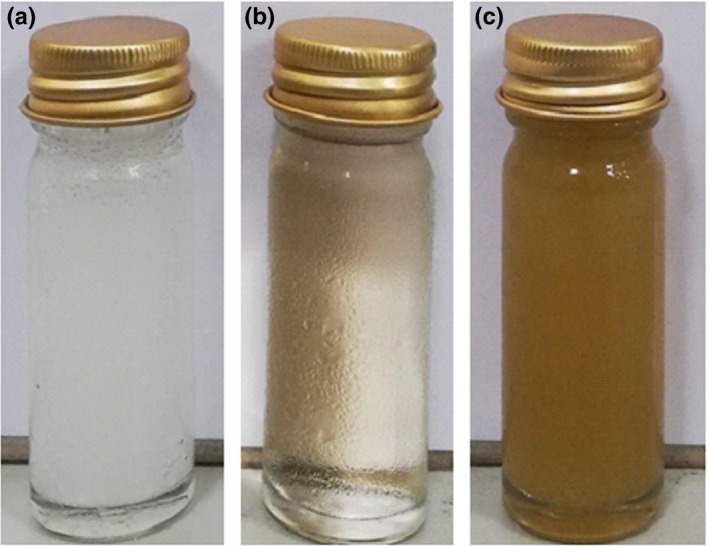
Visual observation of (a) coconut sap, (b) sugar palm juice, and (c) sugarcane juice

The color parameters (L*, a*, b*, C*, and H*) and browning index (BI) for coconut sap, sugar palm, and sugarcane juices are shown in Table 1. In addition, visual observation on the color of these three samples is shown in Figure 1. Visually, coconut sap was lighter in color as compared to sugar palm and sugarcane juice. Coconut sap had significantly high (*p* ≤ .05) L* value and low a* and b* values (Table 1), indicating it exhibits a lighter color which was comparable with previous findings (Hebbar et al., 2015; Jagannadha Rao, Das, & Das, 2009; Naknaen & Meenune, 2015).

### 3.4 Hue angle (H*) and chroma (C*)

The values of hue angle (H*) and chroma (C*) of coconut sap were 1.31 and 3.62, respectively, which were significantly (*p* ≤ .05) lower than sugar palm and sugarcane juices. This indicates coconut sap exhibited a lower color saturation as higher values of H* and C* indicate higher saturation of color.

### 3.5 Browning index

According to Table 1, the value of browning index (BI) for sugar palm juice was the lowest (−13.71), followed by coconut sap (−10.21) and sugarcane juice (18.65). With a lower browning index, coconut sap would be suitable as an ingredient for many food‐related products.

### 3.6 Sugar profile

**Figure 2:**
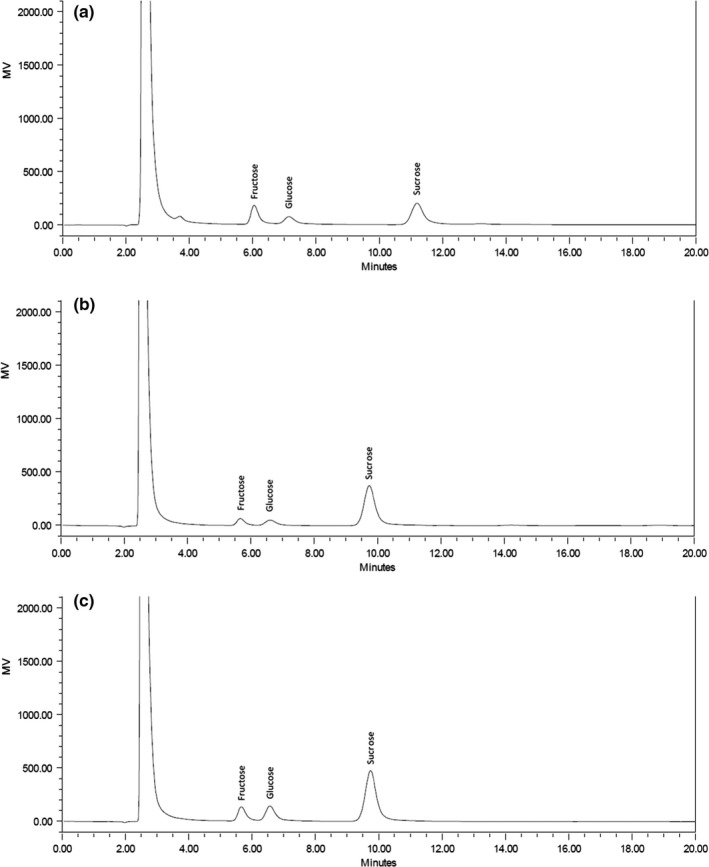
(a) Sugar chromatogram of coconut sap. (b) Sugar chromatogram of sugar palm juice. (c) Sugar chromatogram of sugarcane juice

**Figure 3:**
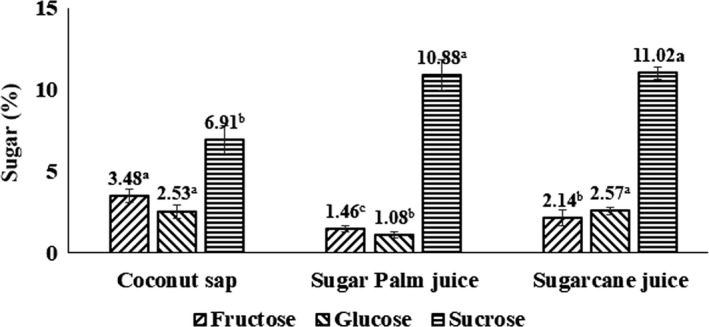
Sugar contents of coconut sap, sugar palm juice, and sugarcane juice

The HPLC profiles of sugar in coconut sap, sugar palm juice, and sugarcane juice are displayed in Figure 2a,b,c, and the values are presented in Figure 3. Three sugars (fructose, glucose, and sucrose) were detected in coconut sap, and the values were 3.48%, 2.53%, and 6.91%, respectively. In this study, sucrose content was found to be the highest, while lower glucose and fructose contents as compared to a previous report by Somawiharja, Purnomo, Wonohadidjojo, Kartikawati, and Suniati (2018), which reported the amounts of sucrose, fructose, and glucose in fresh coconut sap were 1.76%, 5.76% and 4.46%, respectively. On the other hand, the recorded values of sugar palm juice exhibited higher fructose (1.46%) and glucose (1.08%) contents but lower sucrose content (10.88%) compared with fructose (ND), glucose (ND), and sucrose (13.81%) as reported by Veena et al. (2018).

The concentration of fructose in coconut sap was significantly (*p* ≤ .05) higher than sugar palm and sugarcane juices while the amount of glucose was significantly (*p* ≤ .05) higher than sugar palm juice but lower than sugarcane juice. On the other hand, the sucrose content was significantly (*p* ≤ .05) lower compared with sugar palm and sugarcane juices. The total sugar in coconut sap, sugar palm juice, and sugarcane juice was 12.92%, 13.42% and 15.73%, respectively, indicating coconut sap had the lowest total sugar content. This result was comparable with the result of TSS, as coconut sap recorded the lowest TSS value. As a result, it could be concluded that the sugar produced from coconut sap might be useful for diabetic patients, as it would contain lower amount of sucrose and higher amounts of glucose and fructose. Hence, coconut sugar containing higher amounts of fructose and glucose, and lower amount of sucrose is responsible for lower GI value (Saputro et al. (2017).

### 3.7 Vitamin profile

**Figure 4:**
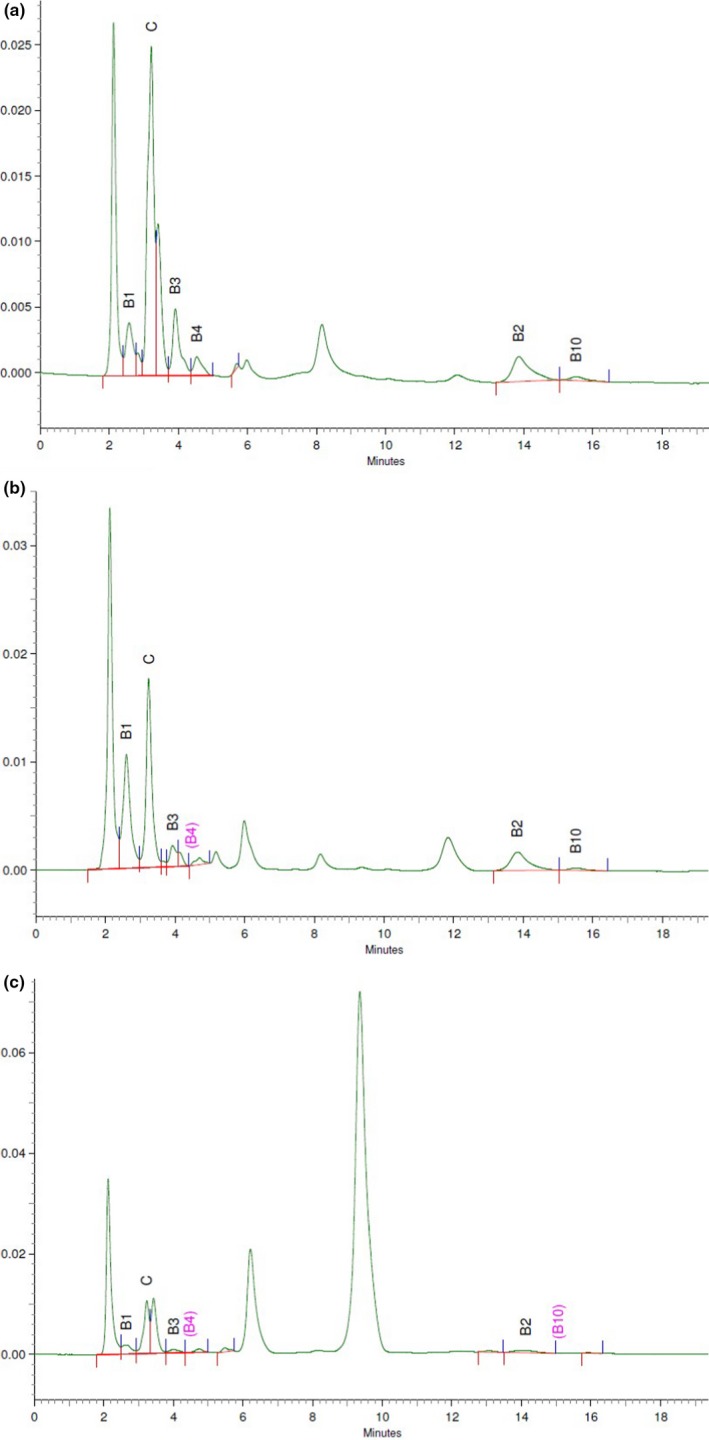
(a) Vitamin chromatogram of coconut sap. (b) Vitamin chromatogram of sugar palm juice. (c) Vitamin chromatogram of sugarcane juice

The HPLC profiles of vitamin in coconut sap, sugar palm juice, and sugarcane juice are shown in Figure 4a,b,c, respectively, and the values are presented in Table 2. Six vitamins (vitamin C, B1, B3, B4, B2, and B10) were detected in coconut sap, and the values were 116.19, 4.33, 1.88, 0.084, 0.53, and 0.33 µg/ml, respectively. In this study, vitamin C, B3, B4, B2, and B10 were found to be significantly higher (*p* ≤ .05) than in sugar palm and sugarcane juices, while vitamin B1 was found to be lower as compared to sugar palm juice but higher than sugarcane juice. Similar amount of vitamin C in coconut sap was reported by Ghosh et al. (2018). Similar amounts of vitamin B1, B2, B3, B4, and B10 were found in coconut sap as measured by (Barh & Mazumdar, 2008; Hebbar et al., 2015). On the other hand, vitamin C and B contents of sugar palm juice were considerably higher than the contents described by Barh and Mazumdar (2008). These differences might be due to different samples used in both studies.

**Table 2:** Vitamin content of coconut sap, sugar palm juice, and sugarcane juice (mg/L)

Vitamins	Coconut sap	Sugar palm juice	Sugarcane juice
C	116.19 ± 0.87^c^	78.24 ± 0.02^b^	42.52 ± 0.00^a^
B1	4.33 ± 0.19^b^	11.73 ± 0.01^a^	2.48 ± 0.01^c^
B3	1.88 ± 0.07^a^	0.63 ± 0.01^b^	0.34 ± 0.01^c^
B4	0.53 ± 0.01^a^	ND	ND
B2	0.084 ± 0.00^a^	0.0738 ± 0.01^b^	0.026 ± 0.00^c^
B10	0.33 ± 0.01^a^	0.24 ± 0.01^b^	ND

Note

Values in the same row sharing different small letters (a, b, c) are expressed as significantly different (*p* ≤ 0.05) among the samples.

Abbreviation: ND, not detected.

The levels of vitamins detected in sugarcane juice were very low. This result was supported by the findings of Nelson and Jones (1930). They found that the sugarcane juice had low vitamin contents. In this study, sugarcane juice contained significantly higher (*p* ≤ .05) vitamin C (42.52 µg/ml) compared with other vitamins. However, vitamin was not detected by Nelson and Jones (1930). In the meantime, Shimizu and Hashizume (1978) found that sugarcane juice only contained vitamin B1, B2, and B6 with low amounts. As a result, it can be concluded that the produced sugar from coconut sap might contain more nutritional values, as it contained high amounts of vitamin C, B1, B3, B4, and B10.

### 3.8 Antioxidant activity

The antioxidant activities of coconut sap, sugar palm juice, and sugarcane juice are shown in Table 3. According to Table 3, the antioxidant activities of coconut sap were measured using DPPH, FRAP, ABTS, and TPC methods (23.42%Sc., 2.09 mM/ml, 21.85%In., and 20.95 mg GAE/100 g, respectively). The amounts of DPPH and TPC found in this study were similar to the values of DPPH (21%) and TPC (20.6 mg GAE/100 g), as reported by Karseno et al. (2018). Besides, antioxidant activities of sugar palm juice were found to be comparable with the value reported by (Nakkaen & Meenune, 2015; DPPH value = 19.46% and TPC value = 64 mg GAE/100 g sample). Antioxidant activities of sugarcane juice expressed as DPPH, FRAP, ABTS, and TPC were 12.40%Sc., 1.19 mM/ml, 12.17%In., and 40.36 mg GAE/100 g sample, respectively. Kadam et al. (2008) studied the antioxidant activities of sugarcane juice and found that DPPH and FRAP were 13.73%Sc. and 0.05 mM/ml, respectively. In the meantime, ABTS and TPC values of sugarcane juice were 28%In. and 60 mg GAE/100 g sample, respectively.

**Table 3:** Antioxidant properties of coconut sap, sugar palm juice, and sugarcane juice expressed using DPPH, ABTS, FRAP, and TPC

Antioxidant properties	Coconut sap	Sugar palm juice	Sugarcane juice
DPPH (Sc. A %)	23.42 ± 0.82^a^	19.82 ± 0.36^b^	12.40 ± 4.59^b^
FRAP (mM/ml)	2.09 ± 0.84^a^	2.56 ± 1.35^a^	1.19 ± 0.48^a^
ABTS (In. A %)	21.85 ± 4.81^a^	17.39 ± 4.10^ab^	12.17 ± 4.33^b^
TPC (mg GAE/100 g sample)	20.95 ± 6.93^c^	54.98 ± 11.12^a^	40.36 ± 12.23^b^

Note

Values in the same row sharing different small letters (a, b, c) are expressed as significantly different (*p* ≤ 0.05) among the samples.

Overall, coconut sap showed significantly higher (*p* ≤ .05) antioxidant activities (DPPH, FRAP and ABTS methods) and significantly lower (*p* ≤ .05) TPC value as compared to sugar palm and sugarcane juices. Coconut sap contained higher amounts of antioxidants; hence, coconut sugar would have better antioxidant properties.

Characterization of phenolic compounds already found from previous literature by (Chen et al., 2011; Lima et al., 2015; Xia et al., 2011) who identified five main phenolic compounds such as gallic acid, protocatechuic acid caffeic acid, p‐coumaric acid, and galangin in coconut sap.

### 3.9 Mineral content

The mineral content for coconut sap, sugar palm juice, and sugarcane juice was presented in Table 4. The major minerals in coconut sap were potassium (960.87 mg/L), sodium (183.21 mg/L), and magnesium (22.91 mg/L). The amount of Ca, Mg, Zn, and Cu in coconut sap was similar to the values reported by Barh and Mazumdar (2008) and Hebbar et al. (2015) which were 0.5, 0.2, 0.018, and 0.1 mg/L, respectively. However, the levels of K, Na, and Fe were lower than values reported by Hebbar et al. (2015) which were 1,461, 690, and 0.5 mg/L, respectively.

**Table 4:** Mineral content of coconut sap, sugar palm juice, and sugarcane juice (mg/L)

Mineral content	Coconut sap	Sugar palm juice	Sugarcane juice
Calcium (Ca)	0.42 ± 0.02^c^	35.43 ± 2.11^b^	200.31 ± 1.30^a^
Magnesium (Mg),	22.91 ± 0.18^c^	37.25 ± 1.99^b^	54.26 ± 1.12^a^
Manganese (Mn),	0.105 ± 0.005^c^	0.379 ± 0.023^b^	1.518 ± 0.343^a^
Copper (Cu)	0.065 ± 0.001^b^	0.180 ± 0.003^a^	ND
Sodium (Na)	183.21 ± 1.42^a^	20.32 ± 0.17^b^	18.20 ± 1.22^c^
Potassium (K)	960.87 ± 12.50^a^	876.66 ± 9.43^b^	770.17 ± 18.20^c^
Zinc (Zn)	0.338 ± 0.002^b^	0.471 ± 0.022^b^	2.45 ± 0.339^a^
Iron (Fe)	1.36 ± 0.038^a^	1.01 ± 0.017^b^	0.33 ± 0.026^c^

Note

Values in the same row sharing different small letters (a, b, c) are expressed as significantly different (*p* ≤ 0.05) among the samples.

Abbreviation: ND, not detected.

The major minerals in sugar palm juice were potassium (876.66 mg/L), calcium (35.43 mg/L), and magnesium (37.25 mg/L). The amount of Mg, Fe, Na, Cu, and Zn was 35.43, 37.25, and 0.47 mg/L, respectively, which were lower than the values reported by Barh and Mazumdar (2008). However, the amounts of K and Ca were higher than the values reported by Barh and Mazumdar (2008) which were 10.7 and 51.2 mg/L, respectively.

The major minerals in sugarcane juice were potassium (770.17 mg/L), calcium (200.31 mg/L), and magnesium (54.26 mg/L). The levels of Ca, Mg, and Na found in this study were higher than the values reported by De Souza et al. (2015). However, the levels of K, Fe, Mn, Zn, and Cu were lower than values reported by De Souza et al. (2015) which were 954.32, 37.80, 6.4, and 2.1 mg/L, respectively.

Coconut sap was found to contain significantly (*p* ≤ .05) higher amount of sodium, potassium, and iron as compared to sugar palm and sugarcane juices. Meanwhile, other minerals in coconut sap were found to be comparable with sugar palm and sugarcane juices. Overall, total minerals in coconut sap were higher than sugarcane and sugar palm juices. These results indicated that a sugar made from coconut sap might also be rich in minerals.

## 4 CONCLUSIONS

The nutritional properties and antioxidant activities of coconut sap were evaluated and compared with those of sugar palm and sugarcane juices. The results showed that coconut sap had high amount of ash (0.27%) with pH of 5.52, but low in TSS (12.14°Brix), indicating coconut sap was rich in mineral with lower acidity and sugar content as compared to sugar palm and sugarcane juices. The coconut sap had lighter color (*L* value) with low browning index, which would be beneficial for food handler in preparation of sugar. Besides, coconut sap was also rich in antioxidants. Coconut sap also contained higher concentration of fructose and glucose with lower concentration of sucrose compared with those of sugar palm and sugarcane juices. Coconut sap also contained higher amounts of vitamins (C, B1, B3, B4, and B10) as compared to sugar palm and sugarcane juices. Hence, coconut sap could be a better potential source for production of healthier sugar.

## CONFLICT OF INTEREST

All the authors declare that there are not any potential sources of conflict of interest. Any interest or relationship, financial or otherwise that might be perceived as influencing an author's objectivity is considered a potential source of conflict of interest. On behalf of all authors, I would like to declare that there is no conflict of interest for our manuscript. This article is original, unpublished and is not being considered for publication elsewhere. All authors have read and approved the manuscript and are aware of submission to Food Science & Nutrition, as research paper. The policy was collectively disclosed to all the authors and they collectively declared that they had no conflict of any financial or otherwise interest with the submission ALL pertinent commercial and other relationships.

## ETHICAL APPROVAL

All the authors were informed, and written consent was obtained and all of them declared their ethical statements before submission of this publication. Ethical Review: “This study does not involve any human or animal testing” and “This study was approved by the institutional Review Board of Unversiti Putra Malaysia (UPM).”

## INFORMED CONSENT

Written informed consent was obtained from all study participants.
